# Appendicitis after endoscopic band ligation for massive ileocecal hemorrhage

**DOI:** 10.1002/deo2.392

**Published:** 2024-05-28

**Authors:** Hiroto Sato, Yu Yamamoto, Akira Kaizuka, Yu Ohtaki, Makoto Toda, Shoichiro Fujishima, Nakao Shirahata, Ryusuke Ae, Takeshi Kanno

**Affiliations:** ^1^ Department of Gastroenterology Yamagata Prefectural Central Hospital Yamagata Japan; ^2^ Division of General Medicine Center for Community Medicine Jichi Medical University Tochigi Japan; ^3^ Department of Gastroenterology Faculty of Medicine Yamagata University Yamagata Japan; ^4^ Department of Gastroenterological Surgery Yamagata Prefectural Central Hospital Yamagata Japan; ^5^ Division of Public Health Center for Community Medicine Jichi Medical University Tochigi Japan; ^6^ R & D Division of Career Education for Medical Professionals Medical Education Center Jichi Medical University Tochigi Japan; ^7^ Division of Gastroenterology Tohoku University Graduate School of Medicine Miyagi Japan

**Keywords:** appendicitis, complication, diverticular bleeding, endoscopic band ligation, ileocecal hemorrhage

## Abstract

A 68‐year‐old man was admitted with hematochezia. Emergency computed tomography showed multiple diverticula throughout the colon. Initial colonoscopy on day 2 showed no active bleeding, but massive hematochezia on day 3 led to the performance of an emergency endoscopy. Substantial bleeding in the ileocecal area obscured the visual field, making it challenging to view the area around the bleeding site. Two endoscopic band ligations (EBLs) were applied at the suspected bleeding sites. Hemostasis was achieved without active bleeding after EBL. However, the patient developed lower right abdominal pain and fever (39.4°C) on day 6. Urgent computed tomography revealed appendiceal inflammation, necessitating emergency open ileocecal resection for acute appendicitis. Pathological examination confirmed acute phlegmonous appendicitis, with EBLs noted at the appendiceal orifice and on the anal side. This case illustrates the efficacy of EBL in managing colonic diverticular bleeding. However, it also highlights the risk of appendicitis due to EBL in cases of ileocecal hemorrhage exacerbated by poor visibility due to substantial bleeding. Endoscopists need to consider this rare but important complication when performing EBL in similar situations.

## INTRODUCTION

Colonic diverticular bleeding (CDB) is the leading cause of lower gastrointestinal bleeding, accounting for around 60% of cases.^1^ Treatments for CDB include endoscopic band ligation (EBL), clipping, application of a coagulation grasper, ligation with a detachable snare, and hypertonic saline‐epinephrine (HSE) injection.^2^ Among these, EBL has gained significant attention in recent years and is considered one of the two major methods for CDB management, alongside clipping. A recent meta‐analysis also revealed that EBL significantly reduces the rebleeding rate within 30 days compared with clipping, further supporting the preference for EBL.^3^ However, a multicenter, large retrospective cohort in Japan revealed EBL‐related complications including diverticulitis (0.16%) and perforation (0.31%).^4^ The authors of that report suggested that mechanical tissue damage from EBL might facilitate bacterial invasion, leading to local inflammation in the form of diverticulitis.^4^ When massive bleeding occurs in patients with CDB, poor visibility complicates the identification of the bleeding sites.^5^ Although CDB can occur at any site along the entire colon, the region extending from the ascending colon to the ileocecum is one of the most common.^1^ Either CDB or appendiceal hemorrhage can theoretically occur in the ileocecal area. When visibility is poor, it may be difficult to determine whether a diverticular hemorrhage is an appendiceal hemorrhage. Although appendicitis can theoretically be a complication of EBL, no cases have been reported to date. We herein present a case of acute appendicitis necessitating emergency surgery after EBL for massive cecal bleeding.

## CASE REPORT

**FIGURE 1:**
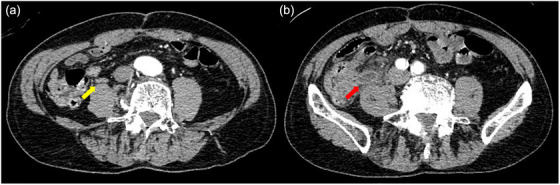
Axial contrast‐enhanced computed tomography images. (a) There is no swelling or thickening of the appendix wall on admission (yellow arrow). (b) Three days after endoscopic band ligation. The appendix exhibited wall swelling and thickening (red arrow)

**FIGURE 2:**
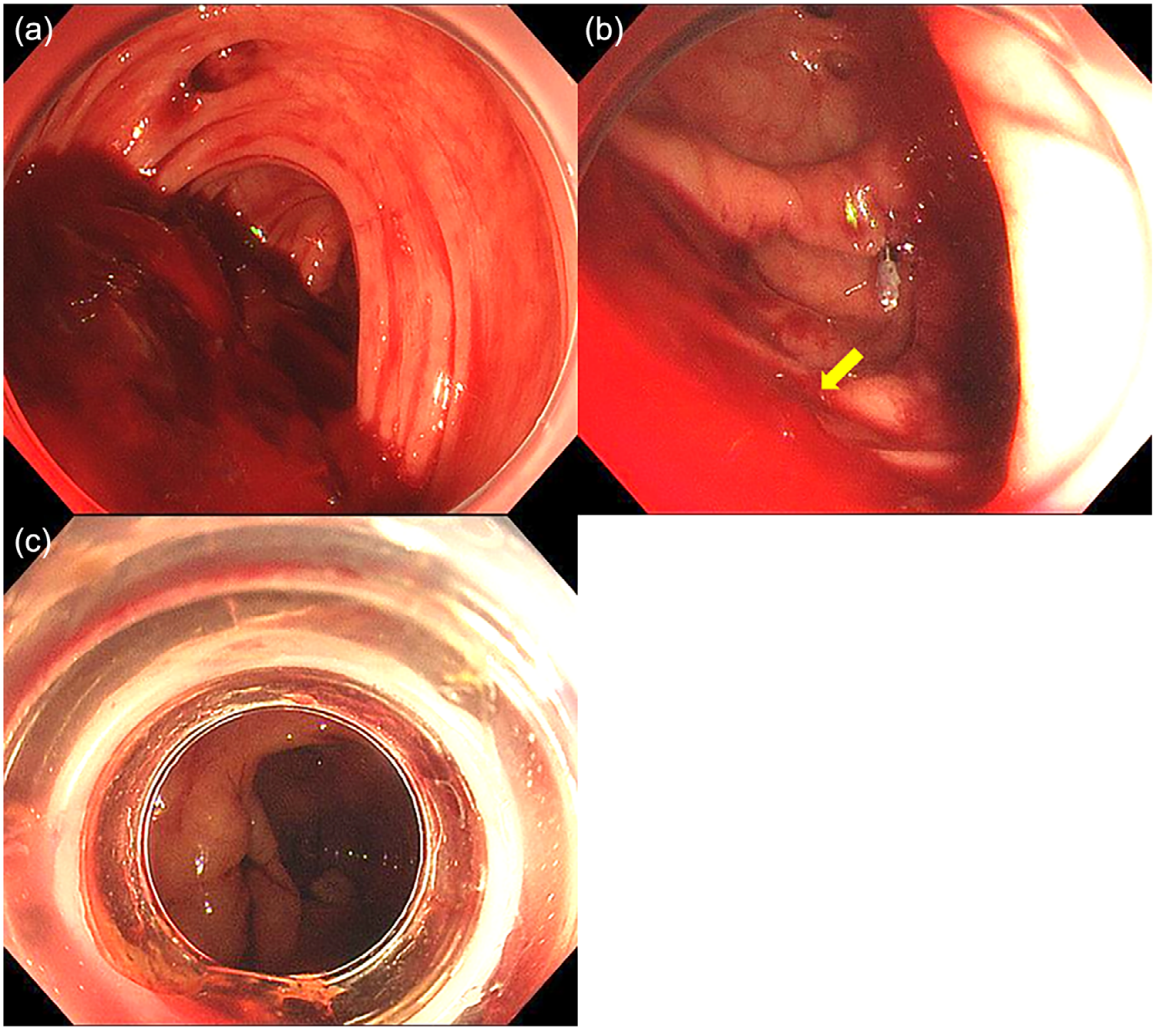
Colonoscopy findings. (a) Ileocecal area with a large amount of clotting blood. (b) A marking clip on the side opposite the suspected bleeding site (yellow arrow). (c) Limited visibility after endoscopic band ligation.

**FIGURE 3:**
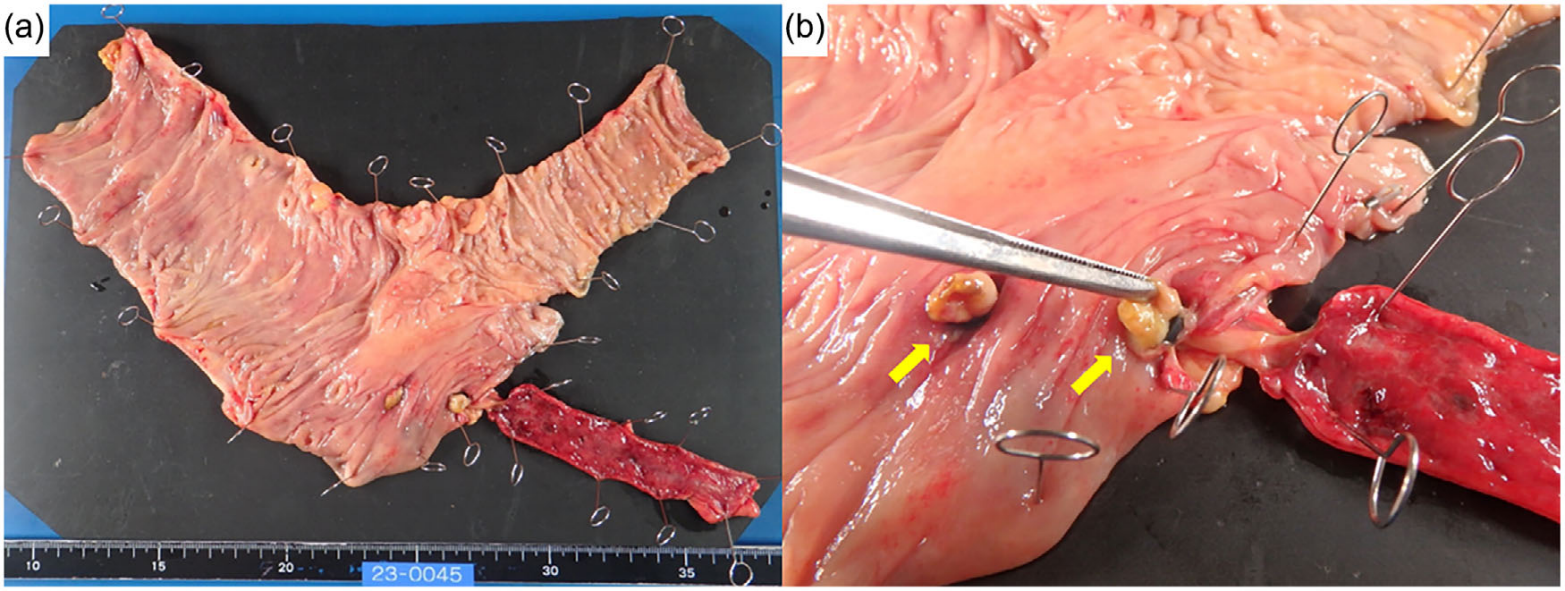
Surgical findings. (a) Overview. (b) Ligations (yellow arrows) on the appendiceal orifice and the anal side.

A 68‐year‐old man was admitted to our hospital with the chief complaint of hematochezia for 2 days. He had a history of open left hemicolectomy for sigmoid diverticular bleeding and was no use of any antithrombotic drugs, steroids, oral hypoglycemic agents, or insulin. On arrival, his blood pressure was 91/59 mmHg, pulse rate was 52 beats/minute, and body temperature was 35.3°C. He had no abdominal pain. His hemoglobin concentration on admission was 7.2 g/dL, white blood cell count was 6590/µL, and C‐reactive protein concentration was 0.12 mg/dL. Emergency contrast‐enhanced computed tomography showed multiple diverticula through the whole colon with no sign of extravasation or no swelling of the appendix wall at that time (Figure 1a). The patient received 8 units of packed red blood cells as conservative treatment during the first 2 days. Initial colonoscopy was performed on day 2 after colonic preparation with polyethylene glycol. Multiple colonic diverticula without active bleeding were observed. Therefore, no hemostatic treatment was performed at the initial endoscopy. However, the patient developed massive hematochezia with a hemoglobin level of 7.2 g/dL on day 3, and an emergency colonoscopy was performed. During the colonoscopy, he suddenly developed a state of shock with a blood pressure of 51/32 mmHg. Therefore, he was transfused with an additional 8 units of packed red blood cells. Because a large amount of blood prevented confirmation of the spatial relationships surrounding the bleeding from an area thought to be a dimple, it proved difficult to determine whether the dimple was the appendiceal orifice or the diverticula (Figure 2a). After placing a marking clip on the side opposite where the blood appeared to be pooling (Figure 2b), we removed the colonoscope and attached the EBL hood (MD‐48910B, Sumitomo Bakelite Co. Ltd., Tokyo, Japan) to the scope. Two EBLs were then performed at the suspected bleeding sites around the marking clip. Hemostasis was successfully achieved; no active gushing bleeding was observed after the EBLs. At this time, the ileocecal valve was visible, but the appendiceal orifice was unclear (Figure 2c). Due to hypotension and limited visibility with the EBL hood, no further detailed observation was performed before the endoscope was removed. After the procedure, the patient's blood pressure rose to 91/50 mmHg. Following endoscopic hemostasis, the patient fasted and no further bleeding occurred; his hemoglobin level was 10.3 g/dL on day 4. Upon resuming food intake on day 6, he developed lower right abdominal pain and a fever of 39.4°C. Urgent computed tomography revealed appendiceal wall thickening and swelling, indicative of inflammation (Figure 1b). Therefore, emergency open ileocecal resection was performed under the diagnosis of acute appendicitis. During the operation, inflammatory changes were evident: the swollen appendix had a fragile root, leading to a minor perforation and subsequent leakage of intestinal juice. The pathological diagnosis was acute phlegmonous appendicitis due to the presence of neutrophil infiltration of the mucosa on the specimen. Two EBLs had ligated the appendiceal orifice and its anal side in the resected pathological specimen (Figure 3a,b) but which was responsible for the initial ileocecal hemorrhage remained unclear. Following surgery, the patient developed transient paralytic ileus, delaying food intake until postoperative day 7. With no further complications, he had a favorable recovery and was discharged on postoperative day 14.

## DISCUSSION

This case demonstrated the emergence of appendicitis as a potential complication after EBL. Theoretically, this complication may be similar to EBL‐associated diverticulitis. Additionally, massive ileocecal hemorrhage with poor visibility may necessitate hemostatic procedures around the appendiceal orifice, highlighting the need to combine different techniques such as HSE, red dichromatic imaging, gel immersion endoscopy, and changing the patient's position to ensure accurate identification of the bleeding source and adequate visibility.

EBL is a hemostatic technique used to control bleeding in patients with esophageal varices and CDB, and it has become a major treatment for CDB. Although several reports have shown no significant difference in the rate of initial hemostasis between EBL and clipping for CDB, the EBL technique can reduce the risk of rebleeding, the need for interventional radiology, and the need for surgical intervention compared with clipping.^5^, ^6^ After EBL for CDB, monitoring for potential complications such as diverticulitis or perforation is warranted,^4^ however, there have been no reported cases of appendicitis. Our patient's operative record and pathological findings suggest that acute appendicitis may have developed as a complication of EBL performed at the appendiceal orifice. In this case, the obstruction of the appendiceal orifice by the ligation may have led to local inflammation, possibly following a mechanism similar to that in which fecal calculus impaction and mucus obstruction are known causes of appendicitis.^7^

Our findings also bring attention to the utility and limitations of endoscopic hemostatic procedures. In this case, whether the vessel responsible for the ileocecal hemorrhage was at the appendiceal orifice or at another site where EBL was performed remains unclear. The large amount of bleeding caused challenges in maintaining clear visibility, complicating the situation and interfering with the precision of the approach. In cases of massive ileocecal hemorrhage where the appendiceal orifice cannot be clearly identified, avoiding EBL may reduce the risk of appendicitis, and interventional radiology could be the suitable alternative treatment. Several possible techniques can be used to improve visibility. First, gel immersion endoscopy may be an effective method; this involves the injection of a clear gel to slow the diffusion of blood and improve the field of view.^8^ This technique can be useful in identifying both the bleeding point and critical anatomical structures such as the appendiceal orifice, even under conditions of significant bleeding. Second, considering that combining HSE injection with clip hemostasis has been reported to enhance hemostasis,^9^ using HSE to control bleeding and visibility prior to EBL may also be a viable strategy. Third, red dichromatic imaging is an image‐enhanced endoscopic technology that enhances the visibility of deep blood vessels and facilitates the differentiation between the bleeding point and the surrounding area.^5^ However, using red dichromatic imaging to differentiate whether the bleeding was from the appendiceal orifice or a diverticulum may have been challenging because it improves the visibility of the bleeding point but lacks detail to examine mucosal texture under the bleeding. Fourth, changing the patient's body position could have improved poor visibility in this case, potentially providing better information about the bleeding site, whether from the appendiceal orifice or other diverticula.

In conclusion, endoscopists should avoid EBL for ileocecal hemorrhage when visibility is poor to prevent unintentional ligation and appendicitis.

## CONFLICT OF INTEREST STATEMENT

None.
